# Development and Testing of a UAV Laser Scanner and Multispectral Camera System for Eco-Geomorphic Applications

**DOI:** 10.3390/s21227719

**Published:** 2021-11-19

**Authors:** Christopher Tomsett, Julian Leyland

**Affiliations:** School of Geography and Environmental Science, University of Southampton, Highfield, Southampton SO17 1BJ, UK; J.Leyland@soton.ac.uk

**Keywords:** Uncrewed Aerial Vehicle (UAV), UAV laser scanning (UAV-LS), multispectral, Structure from Motion (SfM), point cloud, eco-hydraulic, eco-geomorphic

## Abstract

While Uncrewed Aerial Vehicle (UAV) systems and camera sensors are routinely deployed in conjunction with Structure from Motion (SfM) techniques to derive 3D models of fluvial systems, in the presence of vegetation these techniques are subject to large errors. This is because of the high structural complexity of vegetation and inability of processing techniques to identify bare earth points in vegetated areas. Furthermore, for eco-geomorphic applications where characterization of the vegetation is an important aim when collecting fluvial survey data, the issues are compounded, and an alternative survey method is required. Laser Scanning techniques have been shown to be a suitable technique for discretizing both bare earth and vegetation, owing to the high spatial density of collected data and the ability of some systems to deliver dual (e.g., first and last) returns. Herein we detail the development and testing of a UAV mounted LiDAR and Multispectral camera system and processing workflow, with application to a specific river field location and reference to eco-hydraulic research generally. We show that the system and data processing workflow has the ability to detect bare earth, vegetation structure and NDVI type outputs which are superior to SfM outputs alone, and which are shown to be more accurate and repeatable, with a level of detection of under 0.1 m. These characteristics of the developed sensor package and workflows offer great potential for future eco-geomorphic research.

## 1. Introduction

The use of Uncrewed Aerial Vehicles (UAVs) with camera sensors and associated Structure from Motion (SfM) techniques has proliferated in recent years with the development of small, high-endurance aircraft, high-quality lightweight camera sensors, processing software, and increased computer processing power [1]. SfM techniques enable the rapid acquisition of topographic data from a variety of platforms. The versatility of platforms and applications has led to a proliferation of studies within the Earth sciences [2] and beyond [3,4], becoming one of the most widely used high-resolution topographic data collection techniques for characterizing small to medium (10^0^–10^1^ km^2^) areas. In hydrology and fluvial geomorphology, the use of UAVs and SfM has been extensively reviewed [5,6]; however, eco-geomorphic and eco-hydraulic applications have been limited to land cover and vegetation classification mapping exercises based on high-resolution ortho-imagery, rather than derivation of 3D scene characteristics relating to terrain and vegetation structure. The primary reason for this is that SfM alone is limited in its ability to resolve such complex scenes as outlined by Iglhaut, et al. [7]. Below, we provide some background to SfM, identify the related issues with the technique, and list the aims of this research, which seeks to provide a sensor solution capable of overcoming these issues.

SfM relies on the principle that a 3D scene can be constructed from a series of randomly orientated but overlapping photos. SfM uses simultaneous calculations of scene geometry and camera resection in a bundled adjustment technique [2,8], iteratively adding photos to refine the bundle adjustment calculations [9]. These refinements typically apply a least squares minimization method, as there are multiple potential solutions for each set of images [8]. This bundle adjustment process requires the identification of features that can be distinctively tracked between photos, regardless of lighting conditions and camera orientation using variants of Scale Invariant Feature Transform (SIFT) algorithms [2,10]. Despite the attractiveness of SfM as a survey technique, several limitations remain, relating to (i) image quality and overlap and (ii) transformation of a processed cloud into a real-world co-ordinate system. SIFT algorithms use colour gradients, as opposed to absolute pixel values, to determine features in multiple images [9,11]. The number of detectable features identified is directly associated with the output model quality; consequently, poor-quality images may have fewer detectable points and reduce final model quality. Likewise, high image overlap levels are necessary for improving the number of detectable features in multiple images [11]. As a result, good-quality images do not necessarily produce good-quality models, as outlined in Tomsett and Leyland [12].

Prominent features are used to create a sparse point cloud, defined in ‘image space’, which can then be transformed into a chosen coordinate system using Ground Control Points (GCPs) [13]. Dense Multiview Stereo matching (MVS) is then undertaken to produce a highly detailed three-dimensional (3D) model by searching for optimum matches [14]. This process also allows for the removal of areas with higher errors due to the limited matching of features [11]. Transformation of these points relies on good-quality GCPs spread across the study area, capturing changes in elevation in order for a globally optimum solution to be produced [15]. Incorrect GCP placement or reduced GCP accuracy may affect the solving of collinearity equations which can subsequently propagate through the model [13,16]. Assigned GCP locations are then used to perform a seven-parameter transformation, shifting, and rotating the image across three planes, as well as scaling the model [13,17]. The importance of GCPs is self-evident. However recent advances in the miniaturization of high-resolution positioning and orientation sensors, as well as reducing costs, has led to the rise of a new technique which uses ‘direct georeferencing’. The main purpose of direct georeferencing is to enable the collection of 3D data without the need for GCPs. This allows both streamlined in-field data collection and post processing whereby the need for many GCPs is removed [18,19]. However, the technique does not negate the need to include quality controls and checks to ensure data quality.

In addition to the methodological issues outlined above, the suitability and accuracy of SfM can vary substantially, especially in relation to terrain complexity and vegetation cover. While, in principle, low flight heights and high-resolution cameras enable spatial resolutions of up to 0.005 m [20], in practice, model resolutions for bare river reaches are typically between 0.02 and 0.05 m; see [12]. Steep and overhanging terrain reduces the number of detectable points in an image resulting in poor reconstruction in these locations [21]. Vegetation not only reduces the ability to see (i.e., detect and resolve) the study area terrain, it is also not easily or accurately reconstructed by SfM. Both Cook [21] and Dietrich [22] noted that vegetation was sometimes fully captured, but was also often excluded altogether, depending on size and density. UAV SfM is simply not able to resolve the internal structure of vegetation. In relation to an exemplar SfM resolved tree crown, Fawcett, Blanco-Sacristán and Benaud [6] noted that “…the internal structure of this tree could only ever be captured by active laser scanning methods…”. A compounding factor of taller vegetation is shaded areas, particularly in leaf-on conditions. Shaded regions appear homogenous and lack the required features to be detected and matched between image pairs, creating areas of low quality within the final models [23]. Vegetation therefore inhibits the SfM processing workflow, leading to a reduction in quality of the final outputs.

Laser scanning offers a solution to the problems identified above; it is not subject to transformation errors or image quality and overlap related processing errors and it is capable of penetrating complex porous structures such as vegetation, thereby resolving some internal structure and some bare earth terrain points. Terrestrial Laser Scanners have been deployed with great success to elucidate eco-geomorphic processes [24], but they are limited in the scale of application by the need for multiple static setups to capture scenes. UAV-based Laser Scanning (UAV-LS), which combines miniaturized motion sensors and Global Navigation Satellite Systems (GNSS) with lightweight and low-powered laser scanners, has the potential to allow the collection of high-resolution data across relatively large areas. To date, exploration of such systems has been primarily focused on forestry applications [25,26,27], with a few notable exceptions [28,29,30]. However, the off-the-shelf UAV-LS systems used are typically expensive (~GBP 60k–150k for YellowScan or Riegl RiCoptor) and often require ongoing subscriptions to bespoke post-processing software, making them prohibitive in terms of purchase and maintenance costs for many practitioners. Yet the potential for improved data capture, especially in vegetated reaches, set them apart from current survey methods especially in relation to eco-geomorphology.

The potential for improved data capture in vegetated reaches also lends itself to improving spectral data collection. The use of multispectral cameras which obtain imagery at wavelengths beyond the visible spectrum allow a greater understanding of vegetation properties. These have subsequently been used to improve understanding in fields of ecology, coastal monitoring, wildfires, and for above and below water surface analysis [31,32,33,34]. Despite their deployment in several scenarios and increasing efforts to standardise methods between surveys [35,36], there is still more to be done to obtain consistency between surveys.

The aims of this research are to (i) develop a relatively lower cost sensor package using off-the-shelf laser scanning and multi-spectral camera components which are capable of characterizing terrain and vegetation structure; (ii) establish a post-processing technique that is capable of resolving the collected data in real world co-ordinate systems; and (iii) assess the accuracy and repeatability of both UAV-LS and UAV-MS data products against each other and ground check points in the real world, with reference to eco-geomorphic applications.

## 2. Development of a UAV Laser Scanner and Multispectral Camera Sensor System

For the purposes of this paper, references to UAV-LS (UAV Laser Scanning) and UAV-MS (UAV Multispectral) refer to the results of data collection from each sensor, not the entire system.

### 2.1. Components of the System

**Figure 1:**
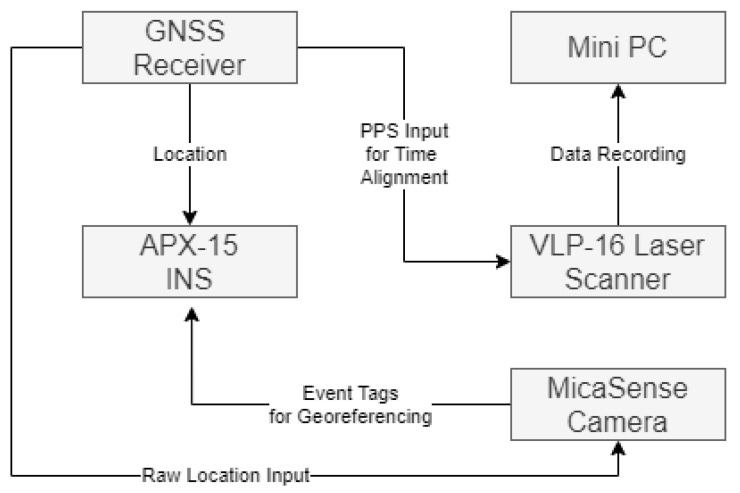
The key components of the developed UAV laser scanner and multispectral camera system for mounting on a UAV platform such as a DJI M600.

The sensor setup is made up of four main constituent parts. The sensor package (a laser scanner and a multispectral camera), an Inertial Navigation System (INS), GNSS input, and a mini-PC for data collection and storage. The complete setup allows for fully georeferenced imagery and point clouds without the need for collecting GCPs. Figure 1 demonstrates how these components are broadly interlinked to provide the datasets required.

The sections below provide detail of each of the sensors and the role they play in the data collection process, commenting on the accuracy of each component of the system.

#### 2.1.1. Applanix APX-15 Inertial Navigation System (INS)

The Applanix APX-15 is a MEMS (micro-electromechanical systems)-based inertial navigation system with a GNSS receiver providing lightweight georeferencing for UAV platforms [37], costing ~GBP 13k in 2017. The MEMS unit uses accelerometers and gyroscopes to resolve both linear movement as well as orientation, collected at a rate of 200 Hz. The unit is light at 60 g and has dimensions of 67 × 60 mm for easy UAV integration. It can interpret 336 GNSS channels over several constellations for high accuracy surveying. After processing, the outputted position and orientation is produced at a rate of 200 Hz for improved georeferencing accuracy. Manufacturer stated positioning is accurate from 0.02–0.05 m with roll and pitch accuracies of 0.025 degrees and heading accuracy of 0.080 degrees after post-processing, values corroborated by Stöcker, et al. [38] when assessing the unit for direct georeferencing.

The unit is capable of outputting a PPS signal (Pulse Per Second) for precise time integration with external devices. This is essential for accurate georeferencing and is an input to the laser scanner to avoid clock drift. In our setup this is set to provide the time on the rising edge of the pulse, in line with the laser scanner specifications (see Section 2.1.2). It also receives event signals to store the exact timings when a multispectral image (see Section 2.1.3) was captured to allow accurate post-processing of images with positional data.

#### 2.1.2. Velodyne VLP-16 Laser Scanner

The Velodyne VLP-16 (puck lite) is a compact form, low-power laser scanner that is optimal for use on UAVs due to its low weight of 590 g [39], costing ~GBP 5k in 2018. The VLP-16 uses 16 laser emitter-detector pairs (903 nm wavelength) which have dual return capability to collect points 360 degrees around the sensor with a viewing angle of 30 degrees. The sensor uses a time-of-flight method to determine distance to an object. It can collect up to 300,000 points per second up to a range of 100 m from the sensor. The scanner has a claimed accuracy of +/− 0.03 m [39], with consistent calibration between units which are stable across a range of temperatures and for long term deployments [40].

The VLP-16 requires a Pulse Per Second (PPS) input to prevent clock drift from start-up totalling around 5 s per day [41]. This equates to roughly 0.07 s by the end of a 20-min flight, and subsequently while flying at 5 ms^−1^ would incur an error of up to 0.35 m. As a result, the APX-15 PPS output is used for accurate synchronisation of clocks between the two sensors.

The VLP-16 outputs data packets for each rotation of the scanner over Ethernet UDP (User Datagram Protocol), which can then be processed in real time or saved [41]. In the current setup, this data is monitored using the free network monitoring software Wireshark and saves a packet capture (.pcap) file to the on-board mini-PC for post fieldwork download and further analysis.

#### 2.1.3. MicaSense RedEdge-MX Multispectral Camera

The MicaSense RedEdge-MX is a five-band multispectral camera, with wavelengths ranging from blue to infra-red (see Table 1), including a red edge band designed to enhance separation between different vegetation characteristics. The camera is compact and lightweight at 230 g and includes a global shutter to decrease distortion and eliminate the need for a gimbal [42]. It cost ~GBP 5k in 2018. Imagery has a ground sampling distance of 8 cm at 120 m and less at the flight heights used in the surveys performed herein (<50 m).

The camera can be triggered in multiple ways. Our setup uses a timer method to capture photos every 1.5 s to maximize forward overlap. This overcomes the shortfall in sidelap due to the relatively narrow field of view of under 50 degrees meaning at flight heights of 50 m, flight path separation of ~23 m is required for attaining 50% sidelap in images. The camera also produces a top of frame output which outputs a signal at the start of image exposure accurate to a few tens of nanoseconds [43], this is then communicated to the APX-15 and recorded as an image event in the flight logs. This provides accurate timestamps from which to extract post-processed position and orientation data for multispectral image processing.

Two methods for obtaining consistent lighting procedures between surveys are used in data capture. First, a manufacturer supplied calibration panel is deployed to adjust for the reflectance values to those that would be expected when imaging the panel. In addition, a Downwelling Light Sensor (DLS) collects data on ambient lighting conditions before writing these to the metadata of each image produced. The DLS is connected directly to the camera and sits on top of the UAV for optimum unimpeded data collection. It is placed away from the other antennae to reduce any interference which may affect the true ambient conditions being recorded. This information is then applied in post-processing to adjust the reflectance values of the images to maintain consistency within the survey and between successive surveys.

#### 2.1.4. Associated Hardware

A Tallysman antenna with L1/L2 capabilities is used to collect GNSS signals across several bands from multiple constellations. The mini-PC is a compact and lightweight Fitlet-i10 (from Fit-PC) which has a low power consumption and runs a basic Windows 10 operating system. This is controlled via an external laptop using a proxy Wi-Fi host and the free TightVNC remote desktop application viewer on the host laptop (https://www.tightvnc.com/, version 2.8.63, accessed on: 17 November 2021). The status of the APX-15 can be checked during flight to monitor the alignment of the INS based on the initialization procedures (see Section 3). The MicaSense camera and recording of the raw VLP-16 data are started before take-off. This also acts as a method to check data in the field and extract data from the sensor setup post field deployment.

### 2.2. Assembly of the System

Each sensor has specific voltage requirements, but we found that 11.1 V satisfied the APX-15, VLP-16, and mini-PC, with an in-line transformer altering the voltage from 11.1 V to 5 V for the MicaSense camera. The setup is powered by a 2650 mAh LiPo battery, which is enough to power the unit for spin up, calibration, survey, cool down, and data download (up to 45 min per charged battery).

**Figure 2:**
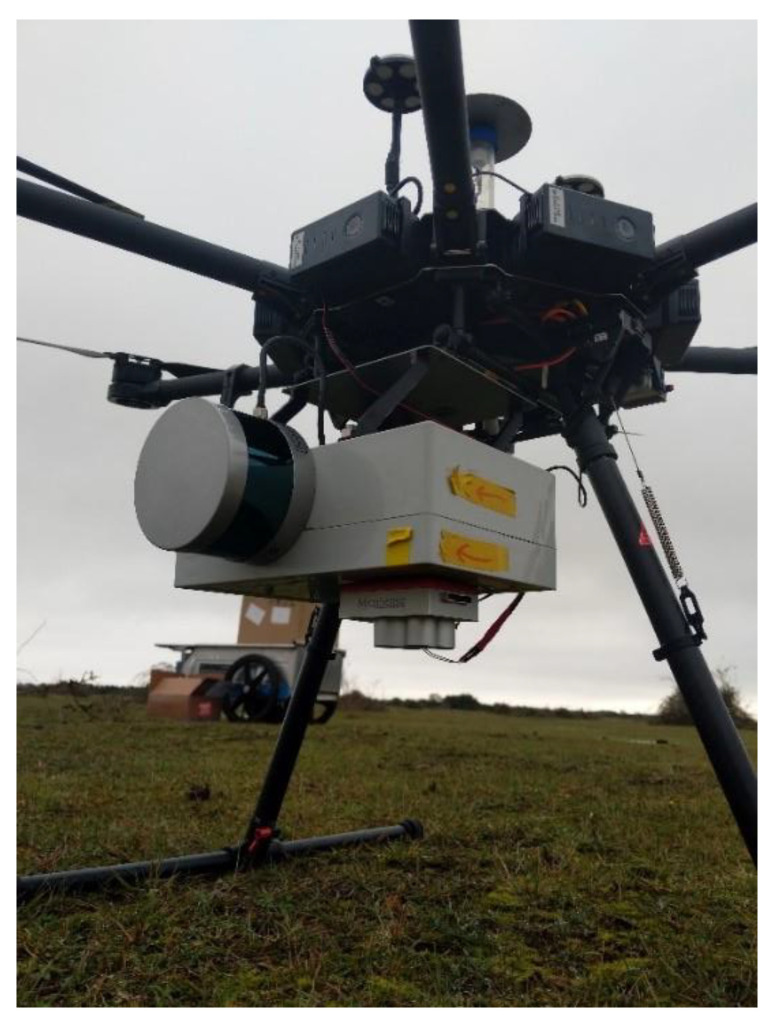
The components of the sensor package assembled within a lightweight plastic box for testing, here mounted on a DJI M600 multirotor platform.

Each component is mounted within or on a lightweight survey box (Figure 2). The VLP-16 is mounted externally with a single screw aligned with two mounting lugs either side for consistent offset calculations, and at a 90-degree pitch angle (forward) scanning across track perpendicular to the flight lines for maximum coverage. The MicaSense camera is mounted with 4 locating screws to keep consistent positioning, as is the APX-15 board so that the X, Y, Z and roll, pitch, and yaw offsets are consistent between flights. The total weight of this survey setup comes in just under 2.5 kg, allowing for flight times of around 20 min using a DJI M600 multirotor aircraft with standard batteries. The unit is then attached on to a DJI mounting plate which sits below the UAV. The mounting plate has dampeners attached between the plate and the UAV to reduce vibrations being passed to the INS, laser scanner, and camera as these are known to have a negative impact on accuracy [44].

Offsets between each of the sensors were measured in the lab before being input into the APX-15 internal memory for processing of each flight. However, as the accuracy of the lab offsets could not be quantified, a calibration procedure has been performed to tighten the offsets before roll, pitch, and yaw adjustments are checked for each survey, as detailed in Section 4.1.4.

## 3. Field Deployment of the System

**Figure 3:**
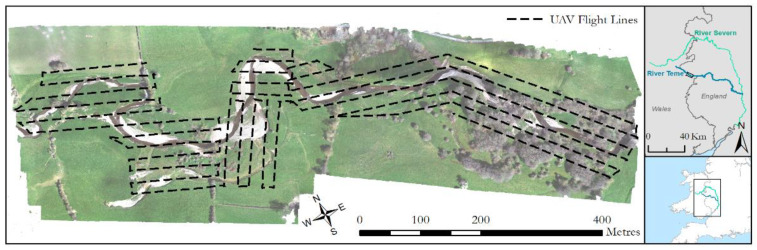
Study site of the River Teme, showing the flight lines used for all surveys from which laser scanning and imagery were captured. Imagery taken from April 2021. Inset shows the location of the study site within the UK and the location of the River Teme, a tributary of the River Severn.

We used a DJI M600 multirotor aircraft for deployment, although in principle the sensor package could be mounted on any airframe that can carry a ~2.5 kg payload and perform the required initialization procedure (see below). For the purposes of this study, data from five separate deployments on a ~1 km reach of the River Teme (Figure 3) made in February, July and September 2020 and April and June 2021 were used. The River Teme is a gravel bed river with alluvial banks that showcases active erosion and deposition processes and exhibits several vegetation types, from grasses to large deciduous trees. A survey-grade Leica GS10 GNSS base station was deployed for each survey, recording raw RINEX observation data for post-processing routines.

The laser scanner and MicaSense camera package collect data in tandem and so flight lines (shown on Figure 3) and speeds (4 m/s) were optimized to provide scan lines for the entire river corridor which delivered at least 50% sidelap and 75% forward overlap for the multispectral imagery. Once powered on, the data from the laser scanner is set to record on the mini PC via a remote desktop connection and the MicaSense is set to run through its setup, including capture of calibration panel images, via a web browser. The APX-15 requires 5–10 min of static data logging both before and after flights to aid forward and backward motion post processing, so is left to stand for this time.

Once ready to fly, the UAV is flown to an elevation of 10 m to perform an initialisation sequence for the INS, consisting of flying at speed forwards, backwards, left, and right to excite and initialise the accelerometers, resulting in a fully aligned heading (confirmed via the INS status). After initialisation, the aircraft is set to auto mode to follow the predetermined flight path (Figure 3). Prior to landing, the initialisation movements of flying forward, backwards, left, and right are performed again to aid in the backwards processing.

## 4. Data Processing Workflow

The following sections outline the workflows developed to process raw field data into spatially referenced products for further analysis. Both the laser scanner and MicaSense multispectral imagery use the same positional raw data, and therefore follow similar initial processing routines.

### 4.1. UAV Laser Scanner and Multispectral Camera Processing

Processing requires three main strands: positional data processing, Velodyne data configuration, and the georeferencing of each data product. The same initial processes to determine position are loosely followed for camera locations when processing multispectral data, with differences to the output data generated.

#### 4.1.1. Inertial Navigation System Processing

Initially, all raw base station data in RINEX format is uploaded to a web service such as AusPos (https://gnss.ga.gov.au/auspos, accessed on: 17 November 2021), whereby the nearest IGS (International GNSS Service) stations are used to correct the position of the base to levels of accuracy as high as 0.02 m (assuming minimum 4 h of raw observations). The reports generated by these services are used to improve the position quality of the original RINEX file by updating the coordinates of the base station or through the download of a new RINEX file with the updated headings depending on the servers being used. This can then be used in the processing of rover data collected by the UAV GNSS receiver.

**Figure 4:**
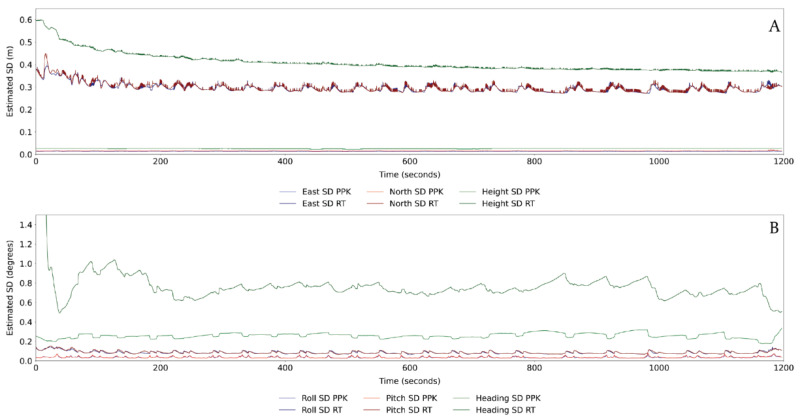
Post-processing results of the Applanix APX-15 data showing how forwards and backwards Kalman filtering reduces errors in the (**A**) X, Y, and Z position of the sensor by an order of magnitude while having a similarly distinct impact on; (**B**) roll and pitch values. Despite relatively larger errors in heading, the post processing helps to minimise the drift in values throughout transects. PPK = Post Processed Kinematic positioning, RT = Real Time positioning, SD = standard deviation.

The positional information from the Applanix APX-15 system is processed using Applanix processing software. This is best done once precise ephemeris data for the satellite is known, to ensure the highest level of precision. Ephemeris accuracy is one of the many factors that can affect the quality of GNSS data being collected. Broadcast ephemeris data initially approximates satellite position, but can be up to 2 h old, whereas precise ephemeris data is based on a monitoring network of ground stations and available at 15 min intervals [45]. Broadcast errors can subsequently be in the range of 1–6 m [45], with precise ephemeris data recommended for cm-level ground-based positioning [46].The post-processed base station is used in conjunction with Applanix IN-Fusion technology [47] to deliver better positioning information when a reduced number of satellites are visible. This routine uses both forward and backward Kalman filtering, designed to estimate unknown variables over time, and uses this to improve positional accuracy [48]. The resultant reported positional accuracy of the sensor package is typically in the range 0.01–0.02 m horizontally and 0.02–0.03 m vertically (Figure 4A). This is assumed to represent the horizontal accuracy of the system in the absence of any field data collected to assess horizontal accuracy. Roll and pitch reported accuracies are within the 0.05–0.10-degree range, with increased standard deviation values during turning; while heading accuracies predominantly vary between 0.2–0.3 degrees, with accuracy decreasing during straight line flight (Figure 4B). This is likely due to slower flying speeds reducing the certainty of the trajectory being calculated. However, the impact of post processing on both the positional and attitudinal data is significant. Improvements in positional location are an order of magnitude improved post processing (Figure 4A), with similar levels of improvement seen in the attitudinal data (Figure 4B). The processed positional data is then exported at a rate 200 Hz (position every 0.005 s) and in the projected coordinate system of choice for combination with the laser scanner and MicaSense camera.

#### 4.1.2. UAV Laser Scanner Raw Data Processing

**Figure 5:**
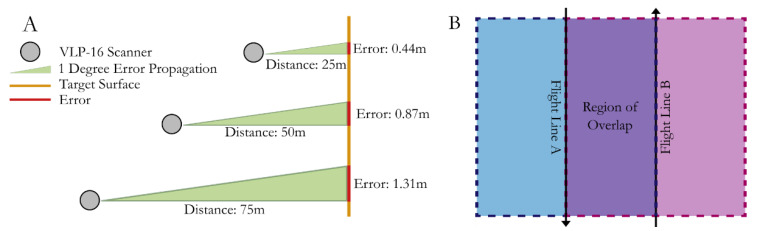
(**A**) Visualisation of how increasing distance from the target surface leads to increasing error associated with any incorrect calculations of roll, pitch, and yaw. The errors calculated are based on a 1 degree miscalculation from a nadir facing laser beam in one direction (i.e., just roll error). (**B**) The region of overlap that is maintained when during cropping to maintain at least two viewing angles of each point within the survey, especially important for vegetated reaches.

The freeware Veloview (https://www.paraview.org/veloview/, version 3.5, accessed on: 17 November 2021) is used to read, display, and output the raw VLP-16 data. Extraneous data from when the platform is initialising on the ground is clipped using the first and last flight line times. Although positional error does not propagate with distance from the sensor, errors in roll, pitch, and heading will (see Figure 5A). Therefore, to limit the effects of this, but to maintain a high ground point density, a filter with maximum distance from the sensor of 50 m in the × and Y planes was applied. Overlapping flight lines in the field were 25 m at maximum, with most flight lines 20–22 m apart, resulting in a minimum of 50% overlap whereby the points from the scan line would be overlapped by at least one other scan (see Figure 5B), but in most cases this would be higher. This overlap maximises point density and accuracy and allows for large extents of the study area to be covered in a single flight. The clipped and filtered data is exported to a series of individual time-stamped ‘scans’ containing a location relative to the sensor centre and intensity. These scans are then ready to be combined with the positional and attitudinal data from the Applanix APX-15.

#### 4.1.3. Combining Laser Scanner and Positional Data

The VLP-16 and Applanix APX-15 positional data are combined using custom python scripts (available at https://github.com/christomsett/Direct_Georeferencing.git, accessed on: 17 November 2021designed to join the two datasets together and produce a final georeferenced point cloud. The workflow accounts for two timing discrepancies that are presented: (i) the VLP-16 references time from the start of the last hour in microseconds, whereas the positional data refers to decimal seconds of the current GPS week; and (ii) there is a discrepancy between UTC time and GPS time, currently 18 leap seconds. Once timestamp discrepancy has been accounted for, a simple join is made based on the nearest time between the two datasets’ time fields. Mismatches in the output rates of both sensors results in a non-perfect synchronization of position and scan data, with a maximum offset in time of 0.0025 s. Surveys are conducted at a maximum speed of 4 ms^−1^, resulting in a maximum error introduced by the time stamp discrepancy of 0.01 m. Based on our data, 80% of all errors due to the timestamp inconsistency are below 0.008 m and 40% are below 0.004 m. Consequently, the relative impact of the timing error is less than the positional accuracy of the system. Next, the scan points are adjusted for the roll pitch and heading of the sensor at the relevant timestamp. A rotation matrix for each point is defined and is then applied to create points relative to the sensor adjusted for roll, pitch, and heading. Finally, points can be transformed using the sensor location into real world coordinates, producing a fully georeferenced point cloud.

#### 4.1.4. Offset and Boresight Angles

To accurately combine the INS and VLP-16 data, it is imperative that accurate offsets and boresight angles are calculated to reduce inconsistencies between surveys [49]. If these are not accurately measured and adjusted, errors will be introduced into the final model [50,51]. As discussed in Section 4.1.2, the relative positional errors of the offsets do not propagate with distance from the sensor. These are initially calculated through the measurements of offsets in the X, Y, and Z planes from a fixed point for each of the INS, VLP-16, and GNSS antenna. These were defined in the lab through handheld measurement and information on sensor dimensions as specified by the manufacturer. This allows the relative positions between each component to be calculated and inputted in to the Applanix software so that the exported positional data accounts for these offsets. The software used also refines these offsets on the fly based on data from the INS, providing an optimal solution. The GNSS antenna is fixed to the UAV via a singular mounting screw, and so is fixed in position each flight. However, while the position of the mounting of the UAV laser scanner sensor system box (Figure 2) is fixed relative to the UAV, the box is subject to small variations in mounting angles between flights.

**Figure 6:**
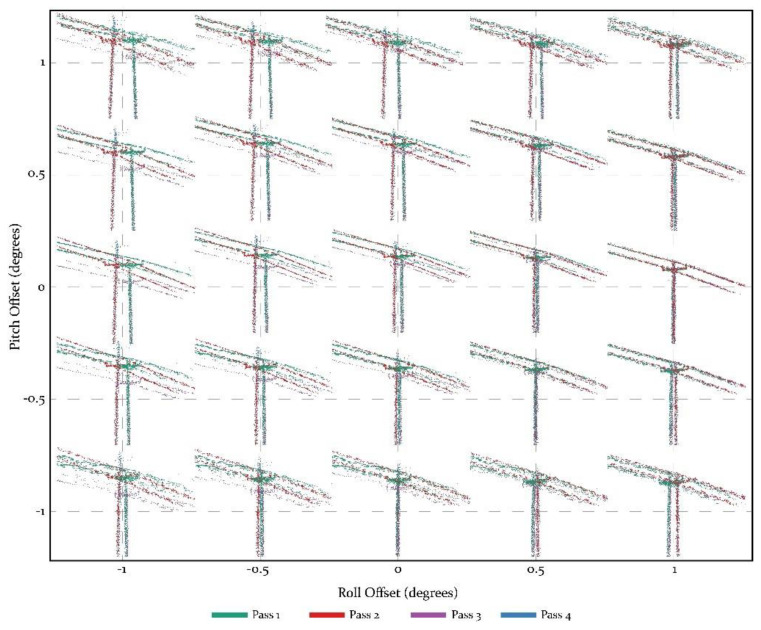
Calculation of offset and boresight angles using an iterative process between multiple passes (from different directions) over an identifiable feature, in this case an electricity pylon.

An iterative process to establish optimum roll and pitch offsets on an identifiable feature (e.g., an electricity pylon) is undertaken for each flight. The initial steps include angles from −1 to 1 in steps of 0.5, before narrowing these down based on visual inspection (Figure 6). As can be seen from Figure 6, this approach allows the identification of trends of change, in this instance moving towards a roll offset of +1 and keeping the pitch offset at 0 degrees resulted in the closest alignment of the pylon and the electricity wires. This is then refined to check for a smaller range of values between the two best combinations of roll, pitch, and heading. In this instance, this evolved testing values of roll between 0.8 and 1.2, and pitch of −0.2 and 0.2. Once an optimum solution has been found, an adjustment is made to alter all roll, pitch, and yaw values before applying the rotation matrix noted in 4.1.3, above. Future work may refine this process to automatically detect the best fit through an ordinary least squares approach.

#### 4.1.5. MicaSense Multispectral Imagery SfM Workflow

To create a position file related to each set of images, custom python scripts (available at https://github.com/christomsett/Direct_Georeferencing.git, accessed on: 17 November 2021) are used to combine the positional tags output from processing the Applanix APX-15 data with each photo taken, accounting for GPS and UTC timing discrepancies between the data. In addition, as each waveband of imagery is suffixed with a 1–5, multiple images have to be given the same positional information, which is written to a database in a format that is readable as reference data for Agisoft Metashape for SfM processing.

Images are loaded in to Agisoft Metashape as a multi camera system, whereby the separate suffixed images are treated as one in the processing workflow, as opposed to attempting to align them separately. Reference data are loaded and assigned to each photo, with accuracy of these coordinates specified as 0.05 m in all three directions and 0.5 degrees in roll, pitch, and heading. This allows for error in position and orientation to be accounted for, as well as any offset or boresight errors introduced to be overcome by the processing workflow. This is in line with the findings noted by Stöcker, Nex, Koeva and Gerke [38], who noted a reduced weighting on the accuracy of external camera orientation led to higher accuracy results in post processing. The photos are then aligned to the highest possible quality to get updated location and orientation of the camera. At this point, the GCPs are used to refine this alignment further; however, the image resolution (~0.035 m) of the multispectral camera introduces some error by impeding precise placement of target centres. Next, a dense point cloud is built alongside mesh and texture surfaces, using high-quality processing settings. These are used to create a tiled model of the study site along with orthomosaics and DEMs at 0.04 m resolution. These models and the dense point cloud are subsequently exported as fully georeferenced datasets.

### 4.2. Data Processing for Comparison and Error Analysis

The processing workflows outlined above result in a UAV-LS-derived point cloud and a MicaSense SfM-generated orthomosaic and point cloud, for each of the five survey dates. Herein, we aim to compare the datasets in two ways: (i) assess the absolute error of the laser scan and SfM data by compared to surveyed ground checkpoints; and (ii) assess relative between-survey accuracy, by comparing areas that experience no morphological change. The latter allows the quantification of a level of detection of change by analysing the repeatability of the surveys between techniques and across survey dates.

#### 4.2.1. Absolute Accuracy Assessment

**Figure 7:**
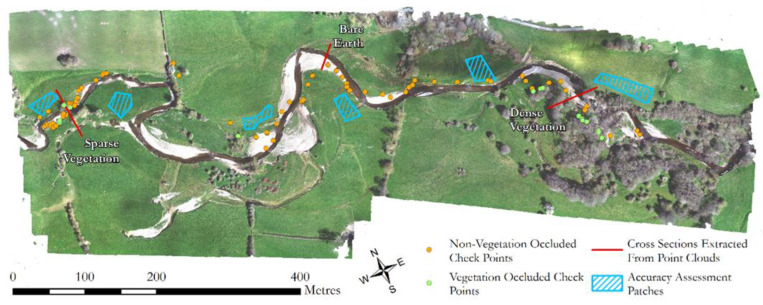
GPS points along the river corridor used for absolute accuracy assessment, including a selection of points which were occluded by vegetation. Highlighted patches are the 6 stable areas used for relative accuracy assessment (see Section 4.2.2). The cross sections relate to those used in the discussion (Section 6.2) to compare UAV-MS SfM and UAV-LS point clouds.

A total of 82 points were collected on 14/04/2021 using a Leica GS10/GS15 base and real-time kinematic rover, including 15 points in locations occluded from overhead view by vegetation (Figure 7). Survey accuracy was assessed by performing cloud to cloud distances (using the open-source CloudCompare software https://www.danielgm.net/cc/, version 2.11.3, accessed on: 17 November 2021) between these point locations and recording the absolute deviation from the measured point in the X, Y, and Z components. Cloud-to-cloud distances were used over point to DEM methods to remove the influence of DEM creation on results and make sure a comparison was made to the most local, observable point.

#### 4.2.2. Relative Accuracy Assessment—Repeatability

Six stable patches (defined as no bare earth change through the survey period) of ground were identified, these being located on areas of land away from the active channel margin. Each patch measured in the range of 690–1350 m^2^ and contained between 680,000 and 1,200,000 points in each of the surveys (approximately 890–950 points per square metre). For each point cloud, the patches were extracted and compared between one another in the following way: (i) between UAV laser scanner and multispectral SfM-derived clouds for the same date; and (ii) between each of the methods individually across all dates. The former helps to compare two methods to derive the same surface, the latter assesses the repeatability of each method. For the purposes of analysis, the patches are combined together and not analysed individually.

## 5. Results

### 5.1. Absolute Accuracy of UAV-LS and UAV-MS (SfM)

For the 82 ground check points, the mean error from the UAV-LS was −0.182 m, with a standard deviation of 0.140 m (Table 2). This implied that the UAV-LS underestimated the distance from the scanner to the surface, with most of the points being above their true ground locations. The average point offset in the combined X and Y plane was 0.012 m, with only 14 (16.5%) points over 0.05 m from the check points. For the same 82 locations, the mean UAV-MS error was −0.469 m and the standard deviation was 0.381 m, suggesting the same direction of error as the UAV-LS. This implies some degree of this error may be due to vertical offset errors during the sensor set up relative to the GNSS receiver on top of the UAV.

For vegetated points, the UAV-LS had a mean error of −0.110 m with a standard deviation of 0.180 m. Although the mean error for vegetated points was lower, the variation in this error was slightly higher. There were higher deviations in the X and Y plane, with an average distance apart of 0.055 m, which is to be expected with a lower below canopy point density, but this appeared to have little effect on the mean error. The UAV-MS appears to perform equally well based on mean error, with a value of −0.181 m; however, the spread of error within vegetated sections is greater, with a standard deviation of 0.572 m and a range of errors over 2 m. This is likely the result of the dense cloud failing to identify the correct depths of points through the canopy, or the ground being obscured completely. This highlights the performance of the UAV-LS method whereby the quality of the resultant point cloud is less diminished by vegetation especially in comparison to the performance of the UAV-MS SfM in heavily vegetated areas.

### 5.2. Relative Accuracy of Surveys: Repeatability

#### 5.2.1. Comparison of UAV-LS and UAV-MS Derived SfM for the Same Dates

**Figure 8:**
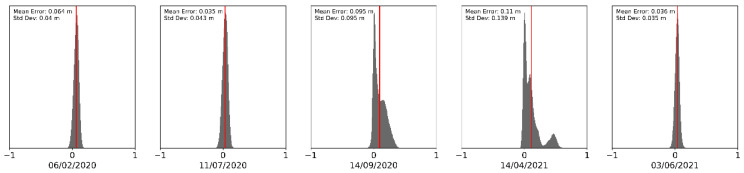
Differences between UAV-LS- and UAV-MS-derived SfM point clouds for the each of the 5 survey dates across the stable patches. Vertical red lines show the position of the mean error in m. This highlights the similarity between both methods for obtaining clouds with some discrepancies on two of the survey dates.

Three of the surveys (February and July 2020 and June 2021) show very good agreement in the data, with mean errors of between 0.03 and 0.06 m, with standard deviations of under 0.05 m (Figure 8). This implies that both the direct georeferencing of the UAV laser scanner and the SfM-derived point cloud are of high quality, with two separate methods obtaining similar results for the same pieces of Earth.

Conversely, there seems to be greater discrepancy in elevations between the methods for surveys in October 2020 and April 2021, showing a greater level of skew in the histograms and multiple peaks. The mean errors are an approximate order of magnitude larger at 0.10 m but more importantly the variation in error increases just as much (Figure 8). Therefore, the confidence in these surveys is lower than others, but are still suitable when investigating morphological change at scales which are typically one and two magnitudes of change larger than this.

#### 5.2.2. Repeatability of Survey Methods: Comparisons across Dates

**Figure 9:**
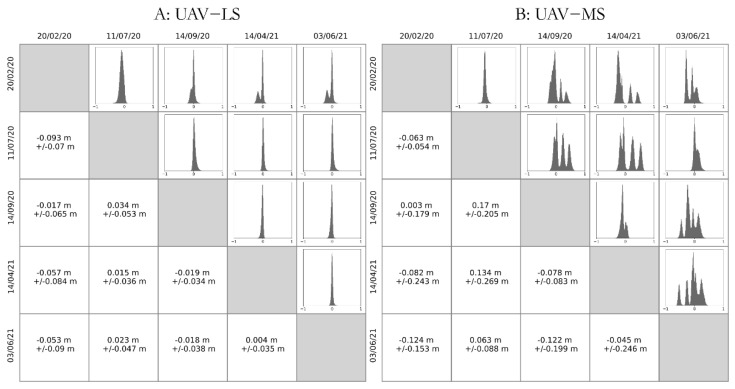
Distributions of errors and mean +/− standard deviation of errors (in m) between surveys for the stable ground patches on different dates for: (**A**) UAV-LS method; and (**B**) UAV-MS-derived SfM methods.

When comparing between the same method on different dates, the first date chronologically is used as the reference cloud, with the latter date the cloud to be compared. As such, a positive difference shows that the latter date points have higher elevation values than the reference (earlier) cloud. Figure 9A shows the comparison of stable ground points between different UAV-LS clouds. The agreement between each pair of surveys is very high, with all mean errors being under 0.1 m and 7 of the 10 combinations having mean errors under 0.05 m. Only comparisons with surveys in February 2020 show any evidence in the histograms of multiple peaks in errors, with all other surveys having a consistent singular peak. These peaks are narrow, with all standard deviations being under 0.1 m, and with most having standard deviations under 0.05 m, suggesting a minimum level of detection of 0.1 m for the UAV laser scanner method when comparing change between two surveys.

In contrast, the error assessment from the UAV-MS derived SfM processing (Figure 9B) suggests that the quality of reconstruction does not present consistent results. The mean errors between pairs of surveys is good, with six of the 10 surveys having values of under 0.1 m; however, the standard deviation of errors is far greater, with seven survey pairs having standard deviations over 0.1 m. This comparison suggests that the errors described in Section 5.2.1 and Figure 8 are likely a result of SfM reconstruction.

**Figure 10:**
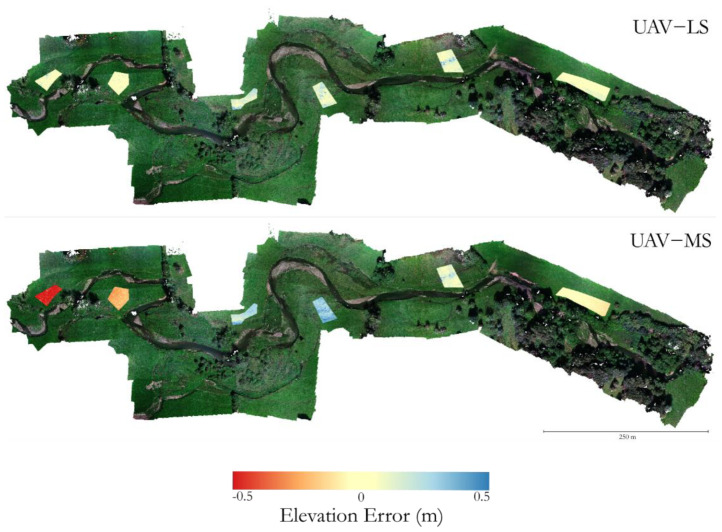
Comparisons of UAV-LS- and UAV-MS-derived SfM methods between April and June 2021 for each of the six stable ground patches, highlighting the spatial variability in accuracy for SfM-derived models compared to UAV-LS methods.

The histograms in Figure 9B show multiple error peaks, suggesting a spatial variation in error across the stable patches. Figure 10 shows a comparison of the Z errors between point clouds from April 2021 and June 2021 for the UAV-LS and UAV-MS datasets in each of the patches. While the UAV-LS data shows a spatially consistent magnitude of error, the UAV-MS appears to show a non-consistent pattern of change, exhibiting both under- and overestimation. It is likely that these patterns are caused not by incorrect location information (i.e., the direct-georeferencing) of the cameras themselves based on the success of the UAV-LS, but rather in the reconstruction and transformation of the image data into the chosen coordinate system. Patches that are close to each other having similar error supports this. This would explain why there is no consistent pattern in the error across survey combinations as seen in Figure 9, as each set of images is processed and fitted optimally, the error produced will be different. This causes the different peaks in errors and for some models to over and under predict elevation in relation to each other.

## 6. Discussion

### 6.1. UAV Laser Scanner and Multispectral System

We assembled a small, <2.5 kg sensor package at a total cost of around GBP 24k (2018 prices), representing a significant saving over commercially available systems, which can cost as much as GBP 150k. UAV-LS absolute accuracy ranged from 0.1 to 0.2 m, but minimum levels of detection based on repeatability comparisons revealed where less than 0.1 m, with most surveys being <0.05 m. These values compare to similar ones reported, for example, by Lin, Cheng, Zhou, Ravi, Hasheminasab, Flatt, Troy and Habib [28] in their coastal application of UAV laser scanning and Jacobs, Hunsaker, Sullivan, Palace, Burakowski, Herrick and Cho [30] for snow depth mapping. By comparison, SfM-derived terrain products showed large errors between surveys, most likely based on variable reconstruction and transformation. Nonetheless, the direct georeferencing and navigation techniques used herein show great promise for future applications and remove the need for many GCPs, instead using just a few as check points with the errors between surveys still less than the magnitudes of change associated with mobile river reaches.

### 6.2. Eco-Geomorphic Applications

**Figure 11:**
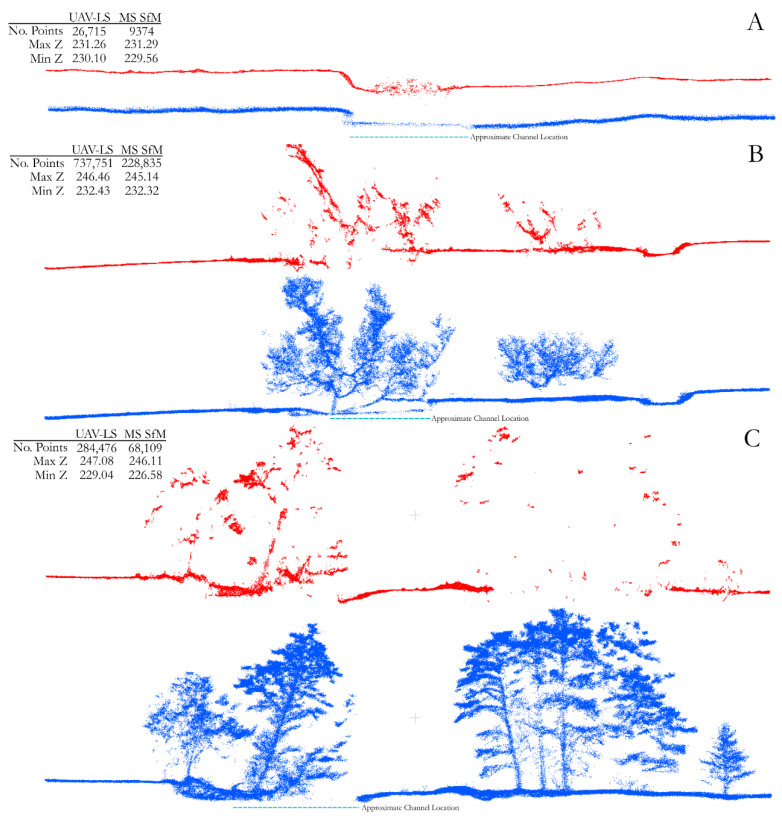
Extracted cross section (see Figure 7 for locations) comparisons of UAV-MS derived SfM point clouds (red) and UAV-LS point clouds (blue) for (**A**) bare earth; (**B**) sparse vegetation; and (**C**) dense wooded vegetation. Statistics show the number of points within each cloud, and the minimum and maximum elevation values.

This paper has focused mainly on the development and application of a UAV-based laser scanning system, but the purpose for developing such a sensor package is for research into eco-geomorphic processes along river corridors. The key innovation in using UAV-based laser scanning instead of (or along with) SfM-derived models is that laser scanning techniques are capable of capturing complex surface features and some measure of vegetation structure [24,29]. It is not within the scope of this study to assess the ability of UAV-LS to collect vegetation structure, nor to derive metrics associated with this. However, Figure 11 provides a visual comparison between UAV-LS and UAV-MS SfM point clouds from three cross sections along the study reach, covering bare earth (A), sparse vegetation (B), and a dense wooded section (C). Despite a drop in point density, the laser scanner better captures the vertical face of the deeply incised river bank in the bare earth cross section, morphology that is smoothed by the SfM reconstruction. SfM is known to struggle at capturing steep banks and overhanging topography due to the reconstruction techniques upon which it relies [21,52], and this can be seen here for the relatively small vertical bank faces of around 1 m shown in Figure 11A.

In addition, the water surface can be identified in both sets of data. Despite the wavelength of the laser scanner (903 nm) being prone to absorption in water, some returns clearly mark out the surface across the bare earth cross section. The SfM-derived results show much higher levels of noise, likely due to surface features and reflection from the moving water increasing the number of erroneous key tie points in the processing steps [53,54,55]. Studies have used the surface reflections of laser scanning to assume a water surface elevation [56], which is more difficult to obtain from SfM techniques [54]. This water surface can then be used to obtain bathymetry by applying refractive corrections to SfM depths in shallow water where the riverbed is visible. A combination of these methods may allow for improved bathymetry from SfM methods by obtaining both subsurface and surface measurements. However, the noise present in this shallow river section highlights the practical difficulties of obtaining bathymetry from imagery.

In the vegetated cross sections, two key observations are made: (i) the vegetation structure is well captured by the UAV-LS; and (ii) the bare earth morphologies of the river and floodplain are equally captured. Despite the variable point density within the canopy from the UAV-LS data, the locations of features such as trunks and low hanging branches are clearly identifiable. This is relevant when considering the impact of vegetation on high flow events where interaction between these features modulates flow patterns. In comparison, the resultant point cloud from the UAV-MS fails to identify many structural elements in the sparse woodland cross section, and even fewer in the dense patches with only a few tree crown points. For studies that aim to observe and quantify vegetation and flow interactions, SfM-based reconstructions have been shown to be unsuitable. In addition, when considering geomorphic change, few bare earth points beneath dense canopies are detected, severely limiting the ability to construct a DEM, with high error potential as outlined in Section 5.1. In the bare earth cross sections (Figure 11A), the cut bank location and angle is fully resolved from the UAV laser scanning data where the SfM reconstruction smooths the feature. There are also several erroneous points in the SfM reconstruction of dense woodland far below the true ground layer (Figure 11C), which would propagate through into increased errors in derived digital terrain models produced from the data. This is likely due to the reduced relative number of points per scan for the SfM-derived clouds as identified on Figure 11. Moreover, any possibility of obtaining water surface or bathymetry data in these vegetated reaches from SfM is removed, yet there is evidence of water surface detection in panel B for the UAV-LS data despite being directly below the canopy. Overall, it would therefore appear that for reaches where there is a large presence of tall, complex vegetation, SfM methods alone are not best suited to eco-geomorphic research.

**Figure 12:**
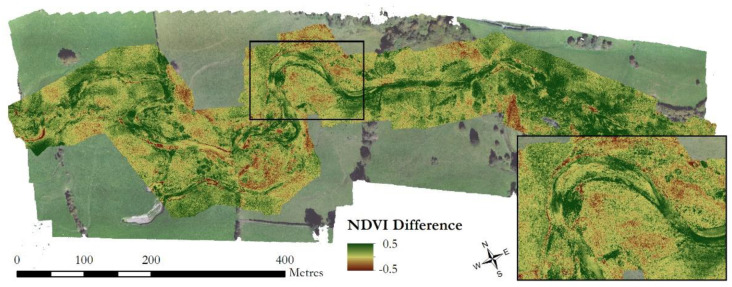
Difference in NDVI values across the study reach between September 2020 and February 2020 surveys. Brown values indicate a decrease in NDVI values and green an increase. Inset shows am enlarged version of a meander and point bar feature, with the large increases in NDVI likely indicating regions of perennial colonisation of sediment deposits by herbaceous pioneer species.

The multispectral data offer some exciting opportunities, for example, making use of the additional near-Infrared wavelength to derive NDVI (Normalised Difference Vegetation Index)-type products linked with the vegetation cover [57]. Such datasets have been shown to improve classifications of vegetation, for example over desert areas [58], and have great potential to be combined with structural data to improve estimation of biomass and vegetation functional type. Measurements of NDVI throughout the year and identification of the magnitude, direction, and temporal rates of change may help in the classification of vegetation. For example, larger changes in NDVI values from winter to summer may indicate the presence of seasonally dependent vegetation. Long-term trends in NDVI can be linked to the underlying fluvial conditions [59], as well as used for monitoring changes in vegetation extent across periods of varying flow conditions [60]. Such research traditionally uses satellite imagery over larger areas, but the advent of compact multispectral systems allows for these interactions to be investigated at the finer spatial resolutions demonstrated herein. For example, Figure 12 highlights the change in NDVI from winter to summer across a bar in our study site, a ~100 m feature over which traditional satellite imagery such as Landsat would capture 1–4 pixels of data. Resolving this level of detail will enable identification of vegetation-flow interactions providing eco-geomorphic insights in addition to those offered from traditional satellite or visible wavelength UAV imagery. While the use of UAV-based multispectral sensing in fluvial research has focussed predominantly on vegetation quality or hydraulic properties such as suspended sediment [61,62], the opportunities to apply methods developed for satellite data at a finer resolution makes combining multispectral rather than traditional RGB imagery with UAV-LS data an exciting prospect for future research.

## 7. Conclusions and Future Work

Having provided a proof-of-concept for the sensor package, and quantified the minimum level of detection at 0.1 m, we have shown that UAV-LS and UAV-MS sensors are capable of delivering high-resolution 3D point clouds and imagery which are able to discretize vegetation structure and spectral response. These methods demonstrate that riparian vegetation can be quantified and analysed at a level of detail that is hitherto unprecedented, capturing additional detail that will allow new insight to be gained in relation to eco-geomorphic interactions. The benefits of obtaining enhanced structural data that cannot be captured by SfM methods alone are evident, and combining the two methods together opens up new avenues of research. For densely vegetated river corridors (and other domains), the benefits of using UAV-LS have been highlighted, with the addition of a multispectral rather than traditional RGB camera allowing additional useful vegetation metrics to be measured.

Future work should seek to use the data to establish metrics which are able to better characterize vegetation function in relation to river corridor evolution. The sensor package developed here, along with comparable commercial units, shows great promise for being able to quantify co-evolving vegetation and geomorphic change trough time, allowing researchers to begin to explore the roles of seasonality, plant maturity and die-back in relation to fluvial dynamics. Furthermore, the potential increase in descriptive functional metrics would be advantageous to machine learning techniques which might seek to link vegetation, geomorphic change, and river flow dynamics through time.

## Figures and Tables

**Table 1:** The band characteristics of the MicaSense RedEdge-MX multispectral sensor.

Band	Wavelength (nm)	Band Width (nm)
Blue	475	32
Green	560	27
Red	668	14
Red-Edge	717	12
Near Infra-Red	842	57

**Table 2:** Absolute errors of each survey method when compared to measured GNSS check points, separated by levels of vegetation obstruction.

Sensor	Category	Summary Statistics				
		Mean Error (Z)	Standard Deviation (Z)	Min	Max	Range
**UAV-LS**	Terrestrial	−0.182 m	0.140 m	−0.366 m	0.424 m	0.790 m
	*Vegetated*	−0.116 m	0.181 m	−0.285 m	0.299 m	0.584 m
**UAV-MS**	Terrestrial	−0.469 m	0.381 m	−1.023 m	1.085 m	2.108 m
	*Vegetated*	−0.181 m	0.572 m	−0.915 m	1.085 m	2.000 m

## Data Availability

The data presented in this study are openly available from Zenodo at: https://doi.org/10.5281/zenodo.5529739 (last accessed: 17 November 2021) in their raw ‘laz’ format. The custom python scripts used to process and combine the VLP-16 and Applanix APX-15 positional data are available at: https://github.com/christomsett/Direct_Georeferencing.git (accessed on: 17 November 2021).
